# Enhanced Potency and Persistence of Immunity to Varicella-Zoster Virus Glycoprotein E in Mice by Addition of a Novel BC02 Compound Adjuvant

**DOI:** 10.3390/vaccines10040529

**Published:** 2022-03-29

**Authors:** Junli Li, Lili Fu, Yang Yang, Guozhi Wang, Aihua Zhao

**Affiliations:** 1 Division of Tuberculosis Vaccine and Allergen Products, Institute of Biological Product Control, National Institutes for Food and Drug Control, Beijing 102629, China; nifdclijunli@163.com (J.L.); flili2007@126.com (L.F.); y923219156@163.com (Y.Y.); tbtestlab@nifdc.org.cn (G.W.); 2 Key Laboratory for Quality Research and Evaluation of Biological Products, National Medical Products Administration (NMPA), Beijing 102629, China; 3 Key Laboratory of Research on Quality and Standardization of Biotech Products, National Health Commission (NHC), Beijing 102629, China; 4 School of Life Science and Biopharmaceuticals, Shenyang Pharmaceutical University, Shenyang 110016, China

**Keywords:** varicella-zoster virus, glycoprotein E, Herpes zoster vaccine, BC02 compound adjuvant

## Abstract

Herpes zoster (HZ) is one of two distinct syndromes caused by Varicella-zoster virus (VZV). A primary infection with VZV causes varicella in susceptible young children. After resolution of the primary infection, VZV establishes a lifelong latency within the cranial or dorsal root ganglia. With increasing age, family history of shingles, immunosuppression or other risk factors, there is a decline in the virus-specific T-cell-mediated immune (CMI) response which allows reactivation of latent VZV in the root ganglia resulting in HZ. There are currently two vaccines that have been approved to prevent HZ and postherpetic neuralgia (PHN) but one is a live attenuated vaccine, the protective effect of which is considered to decrease significantly with the age of the recipient. However, a recombinant subunit vaccine may provide targeted VZV-specific cellular and humoral immunity, giving it a more potent and longer-lasting protective effect against HZ. The current study reports the development of a novel adjuvant, BC02 (BCG CpG DNA compound adjuvants system 02), composed of Al(OH)_3_ inorganic salt adjuvant and BC01 (BCG CpG DNA compound adjuvants system 01), a Toll-like receptor 9 (TLR9) agonist. Immunogenicity and compatibility with recombinant VZV glycoprotein E (gE) in mice were studied. The BC02-adjuvanted gE experimental vaccine was highly effective in eliciting both humoral and cellular immune responses to the recombinant gE glycoprotein and VZV-Oka in a mouse model. The efficient production and long-term persistence of gE and VZV-Oka-specific IFN-γ, IL-2-specific T cells and memory B cells in the early (1W), middle (7W), middle-late (15W), and final (27W) immune stages were established. Results of fluorescent antibody to membrane antigen (FAMA) and serum antibody plaque reduction tests also showed that the BC02 adjuvanted-gE experimental vaccine induced mice to secrete neutralizing antibodies against clinically isolated VZV strains. In combination, the current data suggest that the BC02 compound adjuvant offers a strategy to induce an appropriately strong cellular and humoral immunity against the VZV gE protein subunit to improve vaccine efficacy.

## 1. Introduction

Varicella-zoster virus (VZV) is a neurotropic and lymphotropic alpha-herpesvirus that causes two distinct diseases: varicella and herpes zoster (HZ) [1]. The primary disease of varicella is frequently a benign illness occurring predominantly among susceptible children aged 2–6 years with infection arising largely from respiratory secretions in the acute phase of the patient [2]. HZ occurs when latent VZV in the dorsal root ganglia or cranial nerves undergoes reactivation. The resulting symptoms include rashes, accompanied by severe pain occasionally proceeding to eye involvement, Bell’s palsy and postherpetic neuralgia (PHN), which may persist for weeks, months, or years in adults, significantly compromising health-related quality of life (HR-QOL) [3,4,5]. Deficiency of cell-mediated immunity (CMI), due to the natural process of aging or to immunosuppression, increases susceptibility to HZ severity and PHN development [6,7]. Indeed, the average overall incidence of HZ is estimated to be 3 to 5 cases per 1000 person-years but increases to more than 11 cases per 1000 person-years in people over 80 years of age [8,9]. Moreover, the frequency of recurrent events is higher in immunocompromised individuals, although the incidence of recurrent HZ has not been as well established [10,11].

The optimal treatment of HZ requires early antiviral therapy (acyclovir, famciclovir, valacyclovir) and careful pain management [3]. For patients who have PHN, evidence-based pharmacotherapy using topical lidocaine patch, gabapentin, pregabalin, tricyclic antidepressants (TCAs) or opiates can reduce pain burden [12]. However, in terms of source control, preventive vaccination is the most fundamental and cost-effective means. Currently, there are dozens of HZ vaccines in preclinical or clinical studies but only two have been approved for human use worldwide. Zostavax^®^, a live attenuated zoster vaccine (LZV), contains no less than 19,400 plaque-forming units (PFU) from the Oka/Merck strain of VZV. Produced by Merck, it was the first vaccine licensed for the prevention of HZ and PHN in adults over 50 years of age [13]. The vaccine reduces the incidence of HZ to 69.8% (95% CI 54.1–80.6) in those aged 50–59 years and to 63.9% (95% CI 55.5–70.9) in those aged 60–69 years old [13,14]. However, the magnitude of the VZV-specific CMI response declines over time with the greatest decrease occurring between 6 weeks and 1 year post-vaccination. It has been shown that the VZV-specific CMI response at 6 weeks post-vaccination was significantly lower in vaccine recipients over 70 years than in those 60–69 years old and the protection rate also decreased to 37.6% (95% CI 25.0–48.1) [15,16]. In October 2017, a new adjuvanted recombinant zoster vaccine (RZV), Shingrix^®^ (HZ/su), was approved by the U.S. Food and Drug Administration (FDA) for HZ prophylaxis. The vaccine contains 50 μg recombinant VZV glycoprotein E (gE) and is compatible with liposome AS01_B_ adjuvant [17]. The adjuvant is composed of 3-O-desacyl-4′-monophosphoryl Lipid A (MPL), to activate innate immunity and produce cytokines and Quillaja saponaria Molina, fraction 21 (QS21), to stimulate CD4^+^ and CD8^+^ T cells and antigen-specific antibody responses [18]. Shingrix^®^ demonstrated an overall vaccine efficacy of 97.2% in clinical trials involving participants of 50 years or older, indicating a significantly reduced risk of HZ in these individuals [17]. In addition, Shingrix^®^ displayed similar efficacy in the age group 70–79 years and those ≥80 years showing that there was no decline in efficacy with increasing age and that an age-independent immune response is the most likely protective mechanism. Shingrix^®^ differs from Zostavax^®^ which showed declining efficacy with increasing age [19]. However, the difference is more likely due to potent and longer-lasting CMI by Shingrix^®^. Therefore, adjuvants with synergistic effects on CMI will be the key to the development of protein subunit HZ vaccine.

BC01 (BCG CpG DNA compound adjuvants system 01) was derived from the unmethylated CpG motif-containing DNA fragment from the genome of BCG and exhibits strong adjuvant properties in many species. This biological adjuvant activates innate immune responses through TLR9 receptor-mediated activation of the NF-κB and MAPK signaling pathways [20]. It has recently been shown to have good adjuvant performance in COVID-19 DNA vaccines [21]. Supplementation of BC01 with Al(OH)_3_ inorganic salt adjuvant results in BC02 (BCG CpG DNA compound adjuvants system 02), a second-generation adjuvant system that has the advantage of integrating all components and simultaneously induces Th1 and Th2 immune responses [22,23]. In addition, each component has a synergistic enhancement effect in activating macrophages to participate in the innate immune response in vivo and in vitro [24,25]. As an adjuvant component of the freeze-dried recombinant tuberculosis vaccine AEC/BC02, BC02 is currently in phase I clinical trials (ClinicalTrials.gov Identifier: NCT04239313) and no serious adverse reactions have been found. However, it has not been reported whether the combination of VZV gE with BC02 compound adjuvant still has the advantages described above.

The current study describes the preparation of a compound adjuvant formulation, BC02. Its efficacy as an adjuvant for VZV gE subunit vaccine was evaluated for its ability to elicit CMI, as well as a humoral response, at early and final immune stages in a mouse model. The current data provide a basis for the clinical application of the BC02 compound adjuvant as a HZ vaccine or as another vaccine based on CMI responses as primary protection.

## 2. Materials and Methods

### 2.1. Ethics

Animal experimental protocols were reviewed and approved by the Laboratory Animal Welfare & Ethics Committee of National Institutes for Food and Drug Control (NIFDC). All mice were housed in a temperature- and humidity-controlled chamber with a 12 h light/dark cycle. They were acclimated to the environment for 3 days before experiments, had ad libitum access to food and water and were cared for by specialized staff throughout.

### 2.2. Materials and Reagents

BC01 adjuvant was developed and preserved in our laboratory and Alhydrogel^®^ aluminum hydroxide gel adjuvant was purchased from InvivoGen. IFN-γ, IL-2, and IgG ELISpot kits were purchased from Mabtech. Live attenuated varicella zoster vaccine (lot no. 20201113) and Shingrix^®^, a HZ subunit vaccine adjuvanted with AS01_B_ (lot no. AVZVA031B, AA1BA009), were kindly supplied by the department of respiratory virus vaccines, NIFDC. MRC-5 cell line and clinically isolated strains of VZV were provided by Anhui Longcom Biologic Pharmacy Co., Ltd. Hefei, China. Peptide pool of gE glycoproteins were synthesized by Suzhou Qiangyao Biotechnology Co., Ltd. Suzhou, China. Phosphate-buffered saline (PBS), RPMI 1640 medium, minimum Eagle’s medium, fetal bovine serum (FBS), and 100 × Penicillin-Streptomycin solution were purchased from Gibco. Anti-VZV gE monoclonal antibody (mAb) and FITC-goat anti-mouse IgG (H + L) were obtained from Merk and Beyotime Biotechnology, respectively.

### 2.3. Preparation of Recombinant VZV gE Glycoprotein

The recombinant VZV gE glycoproteins were expressed in Chinese hamster ovary (CHO) cells. Coding sequences were cloned into the pEGC-gE vector containing a CMV promotor and the dihydrofolate reductase gene for CHO cells. Recombinant CHO cells expressing monoclonal gE glycoproteins were selected by limiting dilution analysis (LDA). The gE glycoprotein product secreted into the culture medium was purified by sequential anion-cation exchange, immobilized metal affinity chromatography, and ultrafiltration and analyzed for size, purity, and endotoxin content.

### 2.4. Experimental Vaccine Formulations

To prepare BC02 adjuvanted gE experimental vaccine, 50 µg/mL gE glycoproteins were mixed with 100 µg/mL novel biological adjuvant BC01 and 125 µg/mL aluminum hydroxide adjuvant. For unadjuvanted gE experimental vaccine, 5000 µg/mL endotoxin-free gE protein stock of known molecular weight was diluted with sterile 1 × PBS to 50 µg/mL protein suspension. All vaccine formulations were lightly mixed and placed in a 4 °C thermostatic container for immediate use in mouse immunization.

### 2.5. Immunization and Anatomy of Mice

**Figure 1:**
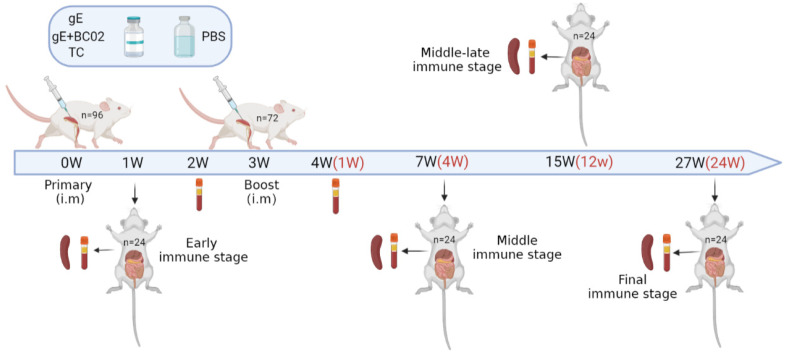
Mouse immunization and sampling schedule.

Eight-week-old specific-pathogen-free (SPF) female BALB/c mice were randomly assigned into groups for intramuscular immunization twice at a 3-week interval. Vaccines were administered to the different groups as follows: un-adjuvanted gE experimental vaccine (5 µg); BC02-adjuvanted gE experimental vaccine (5 µg + 10 µg + 12.5 µg) or PBS (negative control) in a total volume of 100 µL. Test control (TC) was established as an experimental system. Shingrix^®^ was reconstituted by combining gE antigen with AS01_B_ adjuvant according to the manufacturer’s instructions and 1/10 human dose (50 µL) was adjusted to 100 µL with 1 × PBS. At 2 and 4 weeks after the primary immunization (i.e., 1 week after booster immunization), blood was collected from the medial canthal venous plexus of the mice. Mice were sacrificed at 1, 7 (4W), 15 (12W), and 27 weeks (24W) after the primary immunization and peripheral blood and spleen collected (Figure 1). Peripheral blood was allowed to clot at 4 °C for 6 h before centrifugation at 1200× *g* for 10 min at room temperature. Serum was divided into aliquots for storage at −80 °C. Spleens were removed by aseptic dissection and splenic lymphocytes were isolated by density gradient centrifugation after gentle trituration and immunological analysis conducted immediately.

Ninety-six mice were randomly divided into four groups and booster immunizations were performed at intervals of 3 weeks after the primary immunization. Peripheral blood was collected at six different time points, of which four different time points were sacrificed and the spleens were taken and spleen lymphocytes were isolated for immunological detection. TC is the abbreviation of test control.

### 2.6. IFN-γ and IL-2 ELISpot Assays

The numbers of mouse splenocytes producing IFN-γ and IL-2 were determined by ELISpot assays, according to the manufacturer’s instruction manual. In brief, 1.0 × 10^7^ cells/mL single-cell suspensions were prepared from the spleens of immunized mice and 50 µL cells seeded in duplicate in ELISpot plates coated with capture antibodies. Cells were stimulated with gE glycoprotein polypeptide (0.625 µg/mL), Oka strain of varicella vaccine (50 PFU), concanavalin A (0.1 µg, positive control), or culture media (negative control) for 48 h at 37 °C under a 5% CO_2_ atmosphere. IFN-γ and IL-2-secreting cells were detected using an ELISpot kit and positive spots were counted using a CTL-ImmunoSpot^®^ S5 UV Micro Analyzer (Cellular Technology). The total number of spot forming cells (SFC) was calculated by subtracting the number of spots in the culture media wells from the stimulator-added wells and positive irritant Concanavalin A was used for the monitoring of the entire detection system.

### 2.7. Memory B Cells ELISpot Assays

A spleen lymphocyte suspension prepared from mice immunized with experimental vaccine was added to a 46-well plate (1.0 × 10^7^ cells/well) and activated by R848 and rmil-2 stimulation for 72 h in vitro. Activated lymphocytes were washed with RPM1640 medium and the cell concentration was re-adjusted to 2.5 × 10^6^ cells/mL. Total of 100 µL single cell suspension (2.5 × 10^5^ cells/well) was added to each well of an ELISpot plate precoated with glycoprotein gE (20 µg/mL). Cells were cultured for 24 h at 37 °C under a 5% CO_2_ atmosphere and the number of memory B cells secreting gE antigen-specific IgG was counted using a CTL-ImmunoSpot^®^ S5 UV Micro Analyzer (Cellular Technology). The total number of gE-specific IgG antibody secreting cells (ASC) was calculated by subtraction of the medium-cultured control value from that of the stimulated wells.

### 2.8. VZV Fluorescent Antibody to Membrane Antigen (FAMA) Assays

MRC-5 cells were passaged into T25 cell culture flasks and grown into dense monolayers. The growth medium was discarded and an appropriate dose of a clinically isolated strain of VZV was added before incubation for 30 min. Virus growth medium (7 mL) was added and cells were incubated at 37 °C in 5% CO_2_ in air until the cell damage reached 50% to 75% when infected cells were harvested. Infected cells were diluted to a suspension of 1.2 × 10^7^ cells/mL of which 10 µL (12,000 cells/well) was added to each well of a multi-well slide which was placed in a humidified box at 37 °C for 40 min to completely evaporate the water. Infected cells were fixed with 80% acetone for 15 min and 10 µL of a two-fold serial dilution (1:2–1:512) of serum was added dropwise to the corresponding wells. Negative, positive, and serum control wells were also incubated in a humidified box at 4 °C for 12 h. Residual primary antibody was removed and the slides were washed three times with 1 × PBS for 5 min each time. FITC fluorescently labeled secondary antibody was diluted 1:100 in PBS, containing a final concentration of 0.01%. Total of 10 µL Evans blue staining solution was added to each well. After incubation at 37 °C for 1 h in a wet box, slides were washed with 1×PBS as above. A 3 µL volume of 60% glycerol was added dropwise to each well and a coverslip was applied to enable photography under a fluorescence microscope. Serum antibodies were determined to be positive when the serum antibody titer was greater than or equal to 1:4.

### 2.9. Determination of VZV Neutralizing Antibody Titers by Plaque Reduction Test

MRC-5 cells were subcultured into 6-well plates and grown into dense monolayers. A clinically isolated strain of VZV was diluted to 1000 PFU/mL, mixed with equal volumes of serum to be tested at different concentrations (1:2–1:512), and incubated at 37 °C for 1 h. Virus neutralization mixture (100 µL) was transferred to a 6-well plate and incubated at 37 °C and 5% CO_2_ for 1 h. Cell control wells (virus dilution), serum control wells (serum to be tested), and virus control wells (diluted virus) were prepared as control groups. Virus culture solution (3 mL) was added to each well and plates were incubated for 7–10 days to allow formation of virus plaques. Protovirus culture liquid was removed, wells were washed with 1 mL 1 × PBS, and 1 mL Coomassie brilliant blue staining solution was added before incubating for 15 min at room temperature. The remaining dye was rinsed with running water and the number of cytopathic plaques was counted. The neutralizing titers of serum antibodies from immunized mice were defined as the dilution ratio of serum at 50% plaque reduction.

### 2.10. Statistical Analysis

SPSS 16.0 software (IBM) was used for statistical analysis. Differences among experimental groups were analyzed using one-way ANOVA with Tukey’s multiple comparison test. Means and standard errors are expressed as mean ± S.E. and the *p*-values of <0.05 were considered statistically significant.

## 3. Results

### 3.1. Higher Induction of gE-Specific IFN-γ and IL-2 by the Experimental Vaccine with BC02

**Figure 2:**
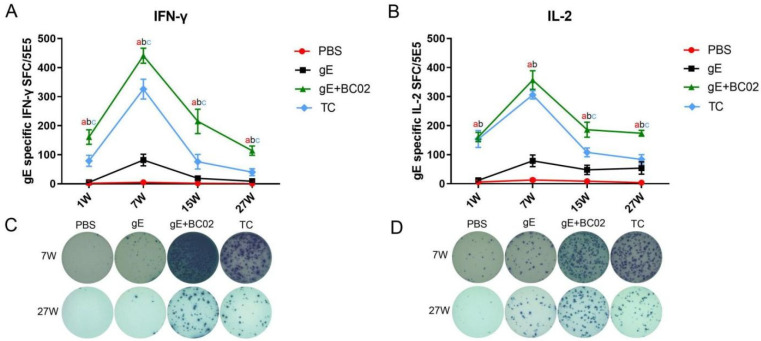
BC02-adjuvanted experimental vaccine induces gE antigen-specific cellular immune response. (**A**,**B**) Mean numbers of gE-specific IFN-γ and IL-2 spot forming cells (SFC) in immunized mice 1, 7, 15, or 27 weeks post immunization determined by ELISpot. Six mice per immunization group. (**C**,**D**) Representative images of gE-specific IFN-γ and IL-2 secreting T cells in mice 7 and 27 weeks post-immunization with BC02-adjuvanted gE glycoprotein. a,b,c: Indicates significant statistical difference between gE+BC02 and PBS, gE or TC, respectively. *p* < 0.05. TC is the abbreviation of test control.

The number of gE antigen-specific IFN-γ cells in spleen lymphocytes of immunized mice was significantly increased. At 1W after primary immunization, there were 161 ± 25 spot-forming cells (SFC) in the BC02-adjuvanted gE experimental vaccine group. Moreover, 441 ± 26 SFC were observed at 7W (4W post-second booster), 215 ± 42 at 15W (12W post-second booster), and 114 ± 16 at 27W (24W post-second booster) in the BC02-adjuvanted gE experimental vaccine group. Negative control (PBS) and non-adjuvanted gE experimental vaccine groups had lower numbers of SFCs than the BC02-adjuvanted gE vaccine experimental group at the above four time points (all *p* < 0.0001, Figure 2A). Numbers of cells secreting gE antigen-specific IFN-γ were 79 ± 19 (1W), 326 ± 34 (7W), 76 ± 25 (15W), and 40 ± 12 (27W) in the test control (TC) group, lower than for the BC02-adjuvanted gE experimental vaccine group (*p* = 0.0072, *p* = 0.0128, *p* = 0.0038 and *p* = 0.0003, Figure 2A).

Numbers of cells secreting gE antigen-specific IL-2 showed an increase and SFC reached a peak at 7W. Numbers of SFC in the BC02-adjuvanted gE experimental vaccine group were 161 ± 16 at 1W, 357 ± 32 at 7W, 186 ± 26 at 15W, and 174 ± 10 at 27W after primary immunization, higher than those of the PBS and non-adjuvanted gE experimental vaccine group at the same time points (all *p* < 0.0001, Figure 2B). SFC in the BC02-adjuvanted gE experimental vaccine group at 1W and 7W were similar to those of the TC group (*p* = 0.9925 and *p* = 0.3250, Figure 2B) and slightly higher by 15W and 27W (*p* = 0.0038 and *p* = 0.0011, Figure 2B).

### 3.2. Higher Induction of Oka-Specific IFN-γ and IL-2 by the Experimental Vaccine with BC02

**Figure 3:**
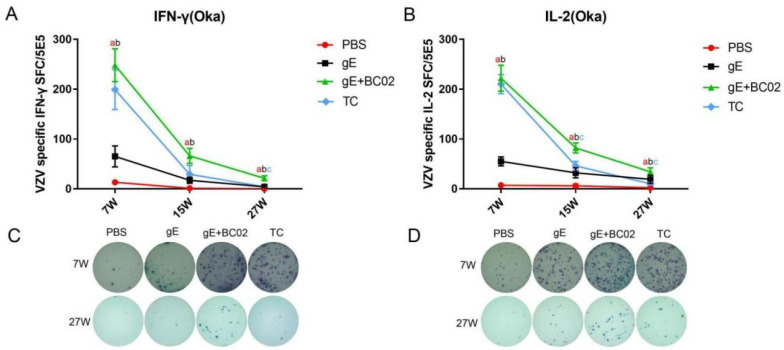
BC02-adjuvanted experimental vaccine induced Oka-specific cellular immune response. (**A**,**B**) Mean numbers of Oka-specific IFN-γ and IL-2 spot forming cells (SFC) in differently immunized mice 7, 15, or 27 weeks post immunization determined by ELISpot. Six mice per immunization group. (**C**,**D**) Representative images of Oka-specific IFN-γ and IL-2 secreting T cells in mice 7 and 27 weeks post-immunization with the BC02-adjuvanted gE glycoprotein. a,b,c: Indicates significant statistical difference between gE+BC02 and PBS, gE or TC, respectively. *p* < 0.05. TC is the abbreviation of test control.

Numbers of Oka-specific IFN-γ SFC formed in the BC02-adjuvanted gE experimental vaccine group were 248 ± 33 (7W), 66 ± 15 (15W), and 21 ± 5 (27W) after the primary immunization. Although SFC showed a decreasing trend over time at all three time points, numbers remained higher than for the PBS group (*p* < 0.001, *p* = 0.0059 and *p* < 0.001, Figure 3A) and the non-adjuvanted gE experimental vaccine group (*p* = 0.0009, *p* = 0.0457, and *p* = 0.0008, Figure 3A) at the same time point. Numbers of Oka-specific IFN-γ cells were also slightly higher than those of the TC group: 199 ± 40 (7W), 29 ± 18 (15W), and 4 ± 1 (27W) with differences being statistically significant at 27W (*p* = 0.0006, Figure 3A). The Oka-specific IL-2 SFC at 7W after immunization in the BC02-adjuvanted gE experimental vaccine group was 222 ± 26, significantly different from that of the PBS control and the non-adjuvanted gE experimental vaccine group (both *p* < 0.001, Figure 3B) but similar to the TC group (*p* = 0.3250, Figure 3B). However, at the other two time points, 15W and 27W, Oka-specific IL-2 SFC were 82 ± 10 and 34 ± 8, respectively, significantly higher than the PBS control, the non-adjuvant gE experimental vaccine, and TC groups (*p* < 0.001, *p* < 0.001, and *p* = 0.0038, Figure 3B) (*p* < 0.001, *p* < 0.001, and *p* = 0.0011, Figure 3B).

### 3.3. Higher Induction of Memory B Cells by the Experimental Vaccine with BC02

**Figure 4:**
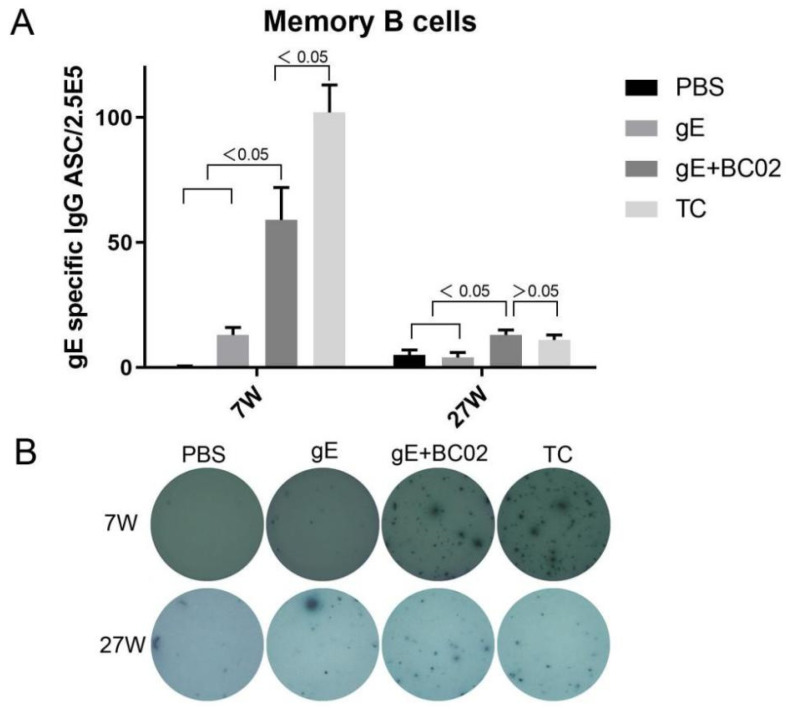
BC02 adjuvant boosted experimental vaccine induced secretion of gE antigen-specific IgG memory B cells. (**A**) Mean numbers of gE-specific IgG antibody secreting cells (ASC) in immunized mice 7 or 27 weeks post immunization determined by ELISpot. Six mice per immunization group. (**B**) Representative images of gE-specific IgG-secreting memory B cells in mice 7 and 27 weeks post-immunization with the BC02-adjuvanted gE glycoprotein. TC is the abbreviation of test control.

Numbers of gE protein-specific IgG memory B cells induced by BC02-adjuvanted gE experimental vaccine were 59 ± 13 at 7W and 13 ± 2 at 27W after primary immunization, significantly higher than those of the PBS group: 0.4 ± 0.2 at 7W and 5 ± 2 at 27W (*p* = 0.0028 and *p* = 0.0267, Figure 4A) and the non-adjuvanted gE experimental vaccine group: 13 ± 3 at 7W and 4 ± 2 at 27W (*p* = 0.0147 and *p* = 0.0209, Figure 4A). However, 7W after primary immunization, the TC group was significantly higher than the BC02-adjuvanted gE experimental vaccine group: 102 ± 11 VS 59 ± 13 (*p* = 0.0208, Figure 4A). By contrast, at 27W, the BC02-adjuvanted gE experimental vaccine group (13 ± 2) was slightly, but non-significantly, higher than the TC group (11 ± 2, *p* = 0.8323, Figure 4A).

### 3.4. Efficient Induction of Anti-VZV Antibody by the Experimental Vaccine with BC02

**Figure 5:**
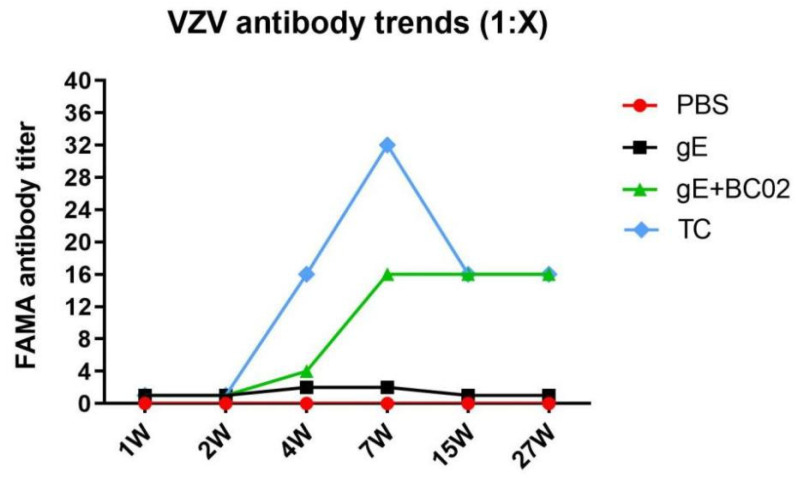
Trend analysis of anti-VZV antibodies induced by BC02-adjuvanted gE experimental vaccine. FAMA serology test to determine susceptibility to VZV infection. An antibody titer cut-off value of ≥1:4 represents seropositivity, indicating potential protection against VZV infection (Sera from 6 mice were pooled). TC is the abbreviation of test control.

Serum from 1W, 2W, 4W, 7W, 15W, and 27W post-primary immunization were collected and changes in anti-VZV antibodies were detected by FAMA assays (Figure 5). Anti-VZV antibody titers of the BC02-adjuvanted gE experimental vaccine group were less than 1:4 at 1W and 2W after primary immunization, so that antibody levels could not be described as seropositive. However, seroconversion of the anti-VZV antibody could be seen to begin at 4W, that is, 1W after the booster immunization. At 7W, anti-VZV antibody titer was 1:16 and remained stable at 15W and 27W. By contrast, in the non-adjuvanted gE experimental vaccine group, all anti-VZV antibody titers remained below 1:2 at the above six time points. It is noteworthy that, at 4W and 7W after the primary immunization, the anti-VZV antibody titer in the TC group was higher than that in the BC02-adjuvanted gE experimental vaccine group, reaching 1:32 at 7W. This indicates an increased level of specific antibodies due to the booster immunization. Over time, antibody levels in the two groups gradually converged. 4W after the initial immunization with BC02-adjuvanted gE experimental vaccine, mouse serum could be diluted 64 times without loss of the fluorescence signal of the FAMA assay which also increased with the extension of the immunization time. Indeed, at 27W, a 256-fold serum dilution failed to extinguish the fluorescence signal (Table 1).

### 3.5. Efficient Induction of VZV Neutralizing Antibody by the Experimental Vaccine with BC02

**Figure 6:**
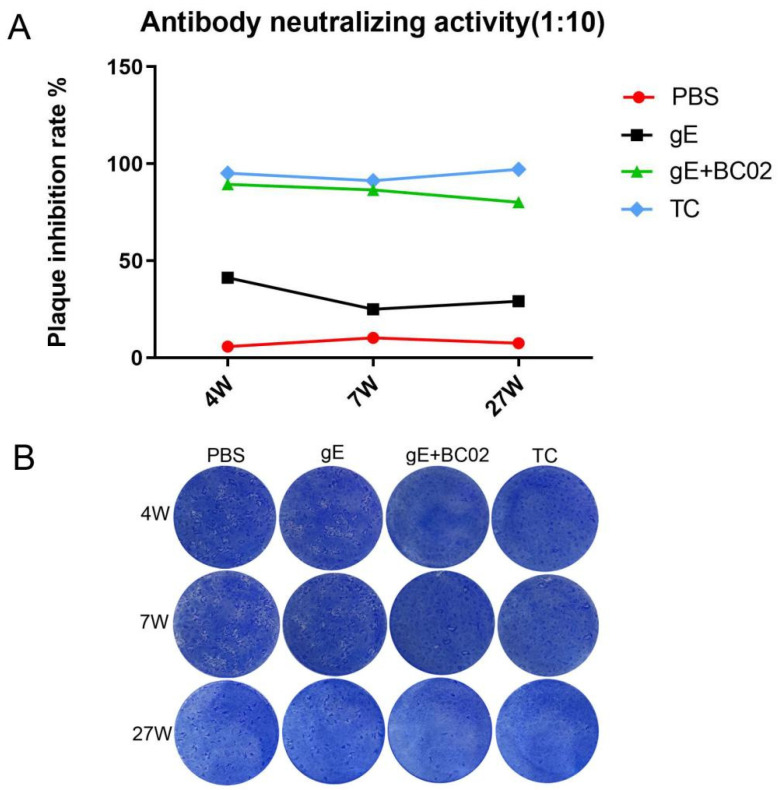
Inhibitory rate of VZV plaque formation by serum antibodies of immunized mice at different time points. (**A**) The immunized mouse serum was diluted at ten-fold and changes in inhibition of VZV plaque formation in different immunization groups with time were measured. (**B**) VZV plaque formation counts in different immunization groups at 4W, 7W, and 27W (Sera from 6 mice were pooled). TC is the abbreviation of test control.

The ability of antibodies from immunized mice to neutralize VZV from clinical isolates showed an inhibition rate of viral plaques in the PBS group of lower than 10% and in the non-adjuvanted gE experimental vaccine group of lower than 42% at 4W, 7W, and 27W. However, inhibition rates of VZV plaque formation were 89.4% at 4W, 86.5% at 7W, and 80.2% at 27W in the BC02-adjuvanted gE experimental vaccine group. These values were slightly, but non-significantly, lower than those of 95.2%, 91.4%, and 97.2% in the TC group (Figure 6A). Results of VZV plaque formation (Figure 6B) show that BC02-adjuvanted gE experimental vaccine and TC immunization mouse serum neutralized clinical VZV isolates. Neutralization effects persisted to 27W post-immunization.

## 4. Discussion

The major viral antigens of VZV are glycoprotein components of the virion envelope, such as glycoprotein E (gE), glycoprotein B (gB), glycoprotein H (gH), and glycoprotein L (gL) [26]. The most abundant and immunogenic of these is considered to be gE (Mr 90–98 kDa) which is expressed on the surface of virus-infected cells and is required for neurovirulence and cell-to-cell transmission in animal models. It has been shown that gE stimulates the production of neutralizing antibodies and also CMI responses and may also contain epitopes recognized by cytotoxic lymphocytes from VZV-infected patients [27,28,29]. In addition, VZV gB, VZV gH, and VZV gL are immunogenic [30,31]. The abundance of VZV gE has led to it being widely developed for use in vaccines or other related detection products [32,33]. The current study expressed recombinant VZV gE glycoprotein in CHO cells and combined it with BC02 compound adjuvant (protected by independent intellectual property rights) as an experimental HZ vaccine. In immunized mice, the hypothesis that the novel adjuvant enhances the strength and durability of specific cellular immune responses was tested to confirm the principle of assisted vaccine protection.

It is generally acknowledged that host reactivity to VZV involves both humoral and CMI responses. The VZV-specific CMI is critical for restricting reactivation and replication of latent VZV and must be considered in HZ vaccine development. Therefore, CMI is considered essential in HZ prevention and in reduction of the severity and incidence of HZ-associated PHN. Clinical studies with Zostavax^®^ have shown that VZV-specific T-cell-mediated immunity, determined by the frequency of IFN-γ-producing cells, is strongly correlated to the protective efficacy of the vaccine [16,34,35]. Three to four weeks after Zostavax^®^ inoculation in healthy adults, multi-functional CD154^+^ IFN-γ^+^ IL-2^+^ TNF-α^+^ cells predominated in the VZV-specific CD4^+^ T-cell population. Furthermore, multi-functional CD4^+^ and CD8^+^ T cells produced significantly higher levels of IFN-γ, IL-2, and TNF-α than mono-functional cells [36]. Multi-functional CD4^+^ T cells were also used as an indicator in clinical trials of the recombinant gE glycoprotein HZ vaccine, Shingrix^®^ [37]. However, the success of RZV is likely to be related to the adjuvant system. Following inoculation with the adjuvanted vaccine, the initial local response is characterized by activation of innate immune cells and production of cytokines. Within hours, both antigen and adjuvant migrate toward the lymph drainage nodes. In this environment, resident natural killer cells and unconventional CD8 T cells are stimulated to produce IFN-γ. Furthermore, dendritic cell recruitment and activation are optimized to present gE to CD4 cells and create a T helper 1 (Th1) polarized environment [38,39,40]. BC02 compound adjuvant with a similar mechanism of action has a wider context of application and has utility for vaccines that require CMI to obtain protection, such as tuberculosis vaccines and HZ vaccines.

The current study reports that the BC02-adjuvanted recombinant gE experimental vaccine enhanced T-cell immune responses in mice and resulted in a significant improvement over traditional aluminum adjuvants which only stimulate humoral immunity. Potent activation of gE- or VZV-specific IFN-γ and IL-2 T cells occurred during the initial stages of immunization, 1W post-primary immunization. T-cell activation reached a peak in the middle-immunization stage, 7 W post-primary immunization (4 weeks post-booster immunization). Although the effector T-cell response decreased with time during the middle-late (15 weeks) and final stages (27 weeks) of immunization, it remained at a higher level compared with the unimmunized and non-adjuvanted gE experimental vaccine-immunized mice. These results demonstrate the necessity of boosting immunization to increase the strength of the activated CMI, and illustrate the reliability and compatibility of the BC02 adjuvant with gE glycoprotein in enhancing immunogenicity. Moreover, the promotion of Th1-type cellular immunity in the early stage of immunization by the BC02 adjuvant demonstrates the enhanced ability of experimental HZ vaccine to prevent VZV reactivation and alleviate PHN. This illustrates one of the reasons why the clinical protection of RZV is higher than that of LZV.

In addition to CMI responses, VZV-specific antibodies can be readily detected in adults with prior varicella and participate in the humoral immune response to HZ vaccine, although the roles of specific antibodies in protection remain unclear. The FAMA test is regarded as the “gold standard” for measurements of protective VZV antibodies due to its high sensitivity and specificity [41,42]. Thus, the absence of antibodies detected by the FAMA test indicates susceptibility to VZV whereas their presence indicates successful immunization. Second, seroconversion, as demonstrated by the FAMA test, is a sensitive and specific indicator of infection. Serial dilutions of sera from immunized individuals are incubated with live VZV-infected human fibroblasts (MRC-5), incubated with fluorescein-tagged immunoglobulin G (IgG) and examined by fluorescence microscopy [43]. A cutoff value of <1:4 serum dilution indicates seronegativity by the FAMA test [44,45] and values ≥ 1:4 indicate protection from varicella, both in naturally infected persons and in healthy vaccinees [46]. FAMA assays of mouse peripheral blood at six time points post-immunization (Figure 1) showed that humoral responses of BC02-adjuvanted recombinant gE glycoprotein reached predicted levels (Figure 5 and Table 1). 4W post-primary immunization (1W post-booster) seroconversion of the anti-VZV-antibody could be observed, reaching a peak during the middle stages of the immune response, 7W post-primary immunization, when the antibody titer reached 1:16. This peak coincided with that of the CMI response described above. Antibody titers were maintained through the middle-late and final stages. Serum from mice which received the non-adjuvanted gE experimental vaccine did not reach the cutoff value of antibody seroconversion during the entire observation period. These results confirm the effectiveness of adding BC02 compound adjuvant to the recombinant gE glycoprotein and that BC02 adjuvant may be superior to humoral immunity in the speed of CMI initiation. Fortunately, although BC02 adjuvant failed to promote the rapid formation of gE antigen-specific neutralizing antibodies in the early stage of immunization, it significantly promoted the strength of this response in the middle stages.

The presence of VZV-specific antibodies in the serum provides a useful indicator of protection against HZ in high-risk persons, whether they have been immunized or naturally infected. Serological tests also reflect the persistence of varicella immunity after vaccination and indicate any waning of vaccine-induced immunity. In addition to FAMA assays, the current study measured virus-neutralizing antibody titers by plaque reduction test. Mice immunized with the BC02-adjuvanted gE experimental vaccine maintained good VZV neutralization ability in the middle (7 weeks), middle-late (15 weeks), and final stages (27 weeks) stages of immunization with plaque reduction rates remaining above 80%. The BC02-adjuvanted gE experimental vaccine stimulated an ability to neutralize the VZV wild strain which was unmatched by the non-adjuvanted gE protein. These results demonstrate that addition of BC02 adjuvant induced the formation of serum antibodies against VZV clinical isolates in mice at a rate which exceeded that of the experimental vaccine without adjuvant. Furthermore, addition of adjuvant maintained the continuous formation of neutralizing antibodies without any reduction in their quality over time. Memory B cells were also seen to peak in the 4th week after booster immunization which was consistent with the trends in antibody formation and serum antibody titers detected by FAMA assays and the plaque reduction test. In addition, the existence of a large number of memory B cells is likely to play an inhibitory role in the recurrence of latent VZV.

In conclusion, the experimental HZ vaccine with BC02 adjuvant recombinant gE glycoprotein induced an earlier Th1-based T-cell immune response which is maintained through the final stages of immunity. Incorporation of the BC02 compound adjuvant enhanced the quality, function, and memory response of the VZV gE glycoprotein-induced humoral immune response, promoted antibody seroconversion, improved antibody neutralization activity, and maintained the persistence of the immune response. The benefits of the addition of BC02 adjuvant to the recombinant gE experimental vaccine were confirmed by the substantial improvement in the immune response elicited. The utility of the BC02 adjuvant for enhancement of the HZ vaccine and its potential for combination with other vaccines that require induction of CMI responses are indicated.

## Figures and Tables

**Table 1:** Fluorescence signal intensity of mouse serum FAMA assay at different time points and different dilution ratios after immunization.

Sample ^1^	Time	FAMA Titration												
		16	32	64	128	256	512		16	32	64	128	256	512
PBS	1W	**−**						7W	**−**					
gE		**+**	**−**						**+**	**+**	**−**			
gE + BC02		**+**	**−**						**+**	**+**	**+**	**+**	**+**	**−**
TC ^2^		**+**	**−**						**+**	**+**	**+**	**+**	**+**	**+**
PBS	2W	**−**						15W	**−**					
gE		**+**	**−**						**+**	**−**				
gE + BC02		**+**	**−**						**+**	**+**	**+**	**+**	**+**	**−**
TC		**+**	**+**	**−**					**+**	**+**	**+**	**+**	**+**	**−**
PBS	4W	**−**						27W	**−**					
gE		**+**	**+**	**−**					**+**	**−**				
gE + BC02		**+**	**+**	**+**	**−**				**+**	**+**	**+**	**+**	**+**	**−**
TC		**+**	**+**	**+**	**+**	**+**	**−**		**+**	**+**	**+**	**+**	**+**	**−**

^1^ Sera from the same group of 6 mice were pooled. ^2^ TC is the abbreviation of test control. Red indicates positive fluorescence signal, green indicates negative fluorescence signal.

## Data Availability

Not applicable.

## References

[1] Zerboni L., Sen N., Oliver S.L., Arvin A.M. (2014). Molecular mechanisms of varicella zoster virus pathogenesis. Nat. Rev. Genet..

[2] Gnann J.W., Whitley R.J. (2002). Clinical practice. Herpes zoster. N. Engl. J. Med..

[3] Cohen J.I. (2013). Clinical practice: Herpes zoster. N. Engl. J. Med..

[4] Gater A., Uhart M., McCool R., Préaud E. (2015). The humanistic, economic and societal burden of herpes zoster in Europe: A critical review. BMC Public Health.

[5] McElhaney J., Gavazzi G., Flamaing J., Petermans J. (2016). The role of vaccination in successful independent ageing. Eur. Geriatr. Med..

[6] Oxman M.N., Levin M.J., Shingles Prevention Study Group (2008). Vaccination against Herpes Zoster and Postherpetic Neuralgia. J. Infect. Dis..

[7] Lang P.O., Govind S., Bokum A.T., Kenny N., Matas E., Pitts D., Aspinall R. (2013). Immune Senescence and Vaccination in the Elderly. Curr. Top. Med. Chem..

[8] Johnson R.W., Alvarez-Pasquin M.J., Bijl M., Franco E., Gaillat J., Clara J.G., Labetoulle M., Michel J.P., Naldi L., Sanmarti L.S. (2015). Herpes zoster epidemiology, management, and disease and economic burden in Europe: A multi-disciplinary perspective. Ther. Adv. Vaccines.

[9] Kawai K., Gebremeskel B.G.G., Acosta C.J. (2014). Systematic review of incidence and complications of herpes zoster: Towards a global perspective. BMJ Open.

[10] Yawn B.P., Wollan P.C., Kurland M.J., Sauver J.L.S., Saddier P. (2011). Herpes Zoster Recurrences More Frequent Than Previously Reported. Mayo Clin. Proc..

[11] Tseng H.F., Chi M., Smith N., Marcy S.M., Sy L.S., Jacobsen S.J. (2012). Herpes zoster vaccine and the incidence of recurrent herpes zoster in an immunocompetent elderly population. J. Infect. Dis..

[12] Johnson R.W., Rice A.S. (2014). Clinical practice. Postherpetic neuralgia. N. Engl. J. Med..

[13] European Medicines Agency. https://www.ema.europa.eu.

[14] Schmader K.E., Levin M.J., Gnann J.W., McNeil S.A., Vesikari T., Betts R.F., Keay S., Stek J.E., Bundick N.D., Su S.-C. (2012). Efficacy, safety, and tolerability of herpes zoster vaccine in persons aged 50–59 years. Clin. Infect. Dis..

[15] Oxman M.N., Levin M.J., Johnson G.R., Schmader K.E., Straus S.E., Gelb L.D., Arbeit R.D., Simberkoff M.S., Gershon A.A., Davis L.E. (2005). A vaccine to prevent herpes zoster and postherpetic neuralgia in older adults. N. Engl. J. Med..

[16] Levin M.J., Oxman M.N., Zhang J.H., Johnson G.R., Stanley H., Hayward A.R., Caulfield M.J., Irwin M., Smith J.G., Clair J. (2008). Varicella-Zoster Virus–Specific Immune Responses in Elderly Recipients of a Herpes Zoster Vaccine. J. Infect. Dis..

[17] Lal H., Cunningham A.L., Godeaux O., Chlibek R., Diez-Domingo J., Hwang S.-J., Levin M.J., McElhaney J.E., Poder A., Puig-Barberà J. (2015). Efficacy of an adju-vanted herpes zoster subunit vaccine in older adults. N. Engl. J. Med..

[18] James S.F., Chahine E.B., Sucher A.J., Hanna C. (2018). Shingrix: The New Adjuvanted Recombinant Herpes Zoster Vaccine. Ann. Pharmacother..

[19] Cunningham A.L., Lal H., Kovac M., Chlibek R., Hwang S.-J., Diez-Domingo J., Godeaux O., Levin M.J., McElhaney J.E., Puig-Barberà J. (2016). Efficacy of the Herpes Zoster Subunit Vaccine in Adults 70 Years of Age or Older. N. Engl. J. Med..

[20] Li J., Fu L., Wang G., Subbian S., Qin C., Zhao A. (2020). Unmethylated CpG motif-containing genomic DNA fragment of Bacillus calmette-guerin promotes macrophage functions through TLR9-mediated activation of NF-κB and MAPKs signaling pathways. Innate Immun..

[21] Zhou Z., Zhang X., Li Q., Fu L., Wang M., Liu S., Wu J., Nie J., Zhang L., Zhao C. (2021). Unmethylated CpG motif-containing genomic DNA fragments of bacillus calmette-guerin improves immune response towards a DNA vaccine for COVID-19. Vaccine.

[22] Chen L., Xu M., Wang Z.Y., Chen B.W., Du W.X., Su C., Shen X.B., Zhao A.H., Dong N., Wang Y.J. (2010). The development and preliminary evaluation of a new Mycobacterium tuberculosis vaccine comprising Ag85b, HspX and CFP-10:ESAT-6 fusion protein with CpG DNA and aluminum hydroxide adjuvants. FEMS Immunol Med. Microbiol..

[23] Zhang Y., Yang Y.C., Zhang J., Bo S.Y., Xin X.F., Wang G.Z. (2011). Immune effect of STAg combined with BCG-DNA and aluminum hydroxide adjuvant in mice. Chin. J. Biol..

[24] Li J.L., Fu L.L., Wang G.Z., Yang X.M., Zhao A.H. (2018). Synergistic enhancement of macrophage innate immune response with BC02 complex adjuvant. Chin. J. Biol..

[25] Li J.L., Fu L.L., Yang Y., Wang G.Z., Zhao A.H. (2022). Analysis of synergistic enhancement of innate immune response by BC02 compound adjuvant components. Chin. J. Biol..

[26] Kutinová L., Hainz P., Ludvíková V., Maresová L., Nĕmecková S. (2001). Immune response to vaccinia virus recombinants expressing glycoproteins gE, gB, gH, and gL of Varicella-zoster virus. Virology.

[27] Forghani B., Dupuis K.W., Schmidt N.J. (1990). Epitopes functional in neutralization of varicella-zoster virus. J. Clin. Microbiol..

[28] Giller R.H., Winistorfer S., Grose C. (1989). Cellular and Humoral Immunity to Varicella Zoster Virus Glycoproteins in Immune and Susceptible Human Subjects. J. Infect. Dis..

[29] Hayward A.R., Burger R., Scheper R., Arvin A.M. (1991). Major histocompatibility complex restriction of T-cell responses to var-icella-zoster virus in guinea pigs. J. Virol..

[30] Forghani B., Ni L., Grose C. (1994). Neutralization epitope of the varicella-zoster virus gH:gL glycoprotein complex. Virology.

[31] Grose C. (1990). Glycoproteins encoded by varicella-zoster virus: Biosynthesis, phosphorylation, and intracellular trafficking. Annu. Rev. Microbiol..

[32] Cohen J.I., Ali M.A., Bayat A., Steinberg S.P., Park H., Gershon A.A., Burbelo P.D. (2014). Detection of antibodies to varicellazoster virus in recipients of the varicella vaccine by using a luciferase immunoprecipitation system assay. Clin. Vaccine Immunol..

[33] Thomsson E., Persson L., Grahn A., Snäll J., Ekblad M., Brunhage E., Svensson F., Jern C., Hansson G.C., Bäckström M. (2011). Recombinant glycoprotein E produced in mammalian cells in large-scale as an antigen for varicel-la-zoster-virus serology. J. Virol. Methods.

[34] Patterson-Bartlett J., Levin M.J., Lang N., Schödel F.P., Vessey R., Weinberg A. (2007). Phenotypic and functional characterization of ex vivo T cell responses to the live attenuated herpes zoster vaccine. Vaccine.

[35] Vermeulen J.N., Lange J.M., Tyring S.K., Peters P.H., Nunez M., Poland G., Levin M.J., Freeman C., Chalikonda I., Li J. (2012). Safety, tolerability, and immunogenicity after 1 and 2 doses of zoster vaccine in healthy adults ≥60 years of age. Vaccine.

[36] Sei J.J., Cox K.S., Dubey S.A., Antonello J.M., Krah D.L., Casimiro D.R., Vora K.A. (2015). Effector and Central Memory Poly-Functional CD4+ and CD8+ T Cells are Boosted upon ZOSTAVAX^®^ Vaccination. Front. Immunol..

[37] Cunningham A.L., Heineman T.C., Lal H., Godeaux O., Chlibek R., Hwang S.-J., McElhaney J.E., Vesikari T., Andrews C., Choi W.S. (2018). Immune Responses to a Recombinant Glycoprotein E Herpes Zoster Vaccine in Adults Aged 50 Years or Older. J. Infect. Dis..

[38] Didierlaurent A.M., Collignon C., Bourguignon P., Wouters S., Fierens K., Fochesato M., Dendouga N., Langlet C., Malissen B., Lambrecht B.N. (2014). Enhancement of Adaptive Immunity by the Human Vaccine Adjuvant AS01 Depends on Activated Dendritic Cells. J. Immunol..

[39] Detienne S., Welsby I., Collignon C., Wouters S., Coccia M., Delhaye S., Van Maele L., Thomas S., Swertvaegher M., Detavernier A. (2016). Central Role of CD169+ Lymph Node Resident Macrophages in the Adjuvanticity of the QS-21 Component of AS01. Sci. Rep..

[40] Coccia M., Collignon C., Hervé C., Chalon A., Welsby I., Detienne S., Van Helden M.J., Dutta S., Genito C., Waters N.C. (2017). Cellular and molecular synergy in AS01-adjuvanted vaccines results in an early IFNγ response promoting vaccine immunogenicity. NPJ Vaccines.

[41] Zaia J.A., Oxman M.N. (1977). Antibody to Varicella-Zoster Virus-Induced Membrane Antigen: Immunofluorescence Assay Using Monodisperse Glutaraldehyde-Fixed Target Cells. J. Infect. Dis..

[42] Iltis J.P., Castellano G.A., Gerber P., Le C., Vujcic L.K., Quinnan G.V. (1982). Comparison of the Raji cell line fluorescent an-tibody to membrane antigen test and the enzyme-linked immunosorbent assay for determination of immunity to varicella-zoster virus. J. Clin. Microbiol..

[43] Williams V., Gershon A., Brunell P.A. (1974). Serologic response to varicella-zoster membrane antigens measured by direct immunofluorescence. J. Infect. Dis..

[44] Breuer J., Schmid D.S., Gershon A.A. (2008). Use and Limitations of Varicella-Zoster Virus–Specific Serological Testing to Evaluate Breakthrough Disease in Vaccinees and to Screen for Susceptibility to Varicella. J. Infect. Dis..

[45] Kim Y.H., Hwang J.Y., Shim H.M., Lee E., Park S., Park H. (2014). Evaluation of a commercial glycoprotein enzymelinked im-munosorbent assay for measuring vaccine immunity to varicella. Yonsei Med. J..

[46] Gershon A.A., LaRussa P., Steinberg S. (1994). Detection of antibodies to varicella-zoster virus using a latex agglutination assay. Clin. Diagn. Virol..

